# Determinants of quality of life in pediatric- and adult-onset multiple sclerosis

**DOI:** 10.1212/WNL.0000000000008667

**Published:** 2020-03-03

**Authors:** Kyla A. McKay, Olivia Ernstsson, Ali Manouchehrinia, Tomas Olsson, Jan Hillert

**Affiliations:** From the Departments of Clinical Neuroscience (K.A.M., A.M., T.O., J.H.) and Learning, Informatics, Management and Ethics (O.E.), Karolinska Institutet; and Centre for Molecular Medicine (K.A.M., A.M., T.O.), Karolinska University Hospital, Stockholm, Sweden.

## Abstract

**Objective:**

To evaluate quality of life (QoL), measured by the EQ-5D, in adults with pediatric-onset multiple sclerosis (POMS) or adult-onset multiple sclerosis (AOMS) and explore determinants of QoL in both groups.

**Methods:**

Data were collected from the nationwide Swedish multiple sclerosis (MS) registry. Demographic characteristics, EQ-5D-3 level, Multiple Sclerosis Impact Scale (MSIS-29) score, Expanded Disability Status Scale (EDSS) score, Symbol Digit Modalities Test score, relapses, and disease-modifying therapy (DMT) exposure were collected on an approximately annual basis (2011–2019). Patients with definite MS with ≥2 EQ-5D measurements collected between ages 18 and 50 were included. The principal outcome was the EQ-5D visual analogue scale (EQ-VAS) score. Linear mixed models compared all available EQ-VAS scores between patients with POMS and patients with AOMS and determinants of EQ-VAS among patients with POMS and patients with AOMS (assessed separately).

**Results:**

A total of 5,094 persons met inclusion criteria: 354 (6.9%) had POMS. A total of 21,357 unique EQ-5D scores were recorded. Most participants were female (70.0%) with a relapsing-onset disease course (98.1%). There was no difference in EQ-VAS scores between patients with POMS and patients with AOMS following adjustment for confounders (β-coefficient for patients with POMS vs patients with AOMS [reference]: 0.99; 95% confidence interval −0.89 to 2.87). Experiencing a relapse, severe neurologic disability (EDSS ≥6.0 vs <3.0), and higher MSIS-29 psychological score were consistently associated with lower QoL, while higher information processing efficiency and exposure to first-line DMTs were associated with higher QoL scores in both groups.

**Conclusions:**

There were no differences in QoL between patients with POMS and patients with AOMS in adulthood. Findings provide support for a focus on reducing neurologic disability and improving psychological status as approaches to potentially improve the QoL of persons with MS.

Multiple sclerosis (MS) is a chronic, unpredictable disease that can affect mobility, cognition, and mood, and has consistently been associated with detriments to quality of life (QoL).^1^ Disease onset typically occurs in early adulthood,^2^ but for fewer than 10% of cases, it arises in childhood.^3^ On average, patients with pediatric-onset MS (POMS) reach disability milestones at a significantly younger age than their adult-onset counterparts, and they appear to be vulnerable to heightened inflammation early in disease^4^ and increased cognitive impairment.^5^ Qualitative research has suggested that a POMS diagnosis is particularly difficult for patients and their families to process.^6^ Few studies have quantitatively assessed the longer-term effect of a pediatric onset of MS on QoL through adulthood. Using Swedish nationwide data, we aimed to evaluate QoL, measured by the EQ-5D, in persons with POMS in comparison to persons with adult-onset MS (AOMS), and explore potential determinants of QoL in both groups. We hypothesized that persons with POMS would experience greater impairments to QoL than persons with AOMS.

## Methods

### Data sources and study population

Data were collected from the Swedish MS Registry (SMSreg), a nationwide quality register that is estimated to capture 80% of all MS cases in Sweden.^7^ Demographic and MS-specific clinical information are collected by neurologists and nurses from persons that attend any neurology clinic in Sweden and include sex, date of MS onset and diagnosis, clinical course, disease-modifying therapy (DMT) exposure, and region of residence in Sweden.

To be included in this cohort study, persons must have been registered in the SMSreg with a definite MS diagnosis. Following recommendations from the International Pediatric MS Study Group, pediatric-onset was defined as having a first recorded clinical symptom of MS prior to the age of 18.^8^ Adult-onset cases included all persons with an MS onset on or after the age of 18 years. To ensure a comparable age and disease duration range between groups, only persons who completed the EQ-5D between ages 18 and 50 and had a disease duration of less than 35 years were included. Participants were required to have a minimum of 2 complete EQ-5Ds recorded. Baseline was considered the date of the first EQ-5D assessment.

### Outcomes and exposures

The EQ-5D-3L^9^ was introduced across Sweden as a component of the Immunomodulation and MS Epidemiology study, a postmarketing phase 4 surveillance study of newly introduced second-line DMTs in Sweden in September 2011. It was adopted as a component of routine care of persons with MS and completed on an approximately annual basis. The EQ-5D is a patient-reported measure of health status, relating to 5 dimensions of health (mobility, self-care, usual activities, pain/discomfort, and anxiety/depression) and a visual analogue scale (referred to as EQ-VAS). Persons are asked to report on each health dimension as having no problems, some problems, or extreme problems. Responses to these 5 questions can be converted into a single index value. The index values are anchored in 1 (full health) and 0 (death), but the scale also allows for negative values, representing states worse than death. The EQ-VAS is similar to a thermometer, on which persons score their perceived health from 0 (worst imaginable health state) to 100 (best imaginable health state).^9^

Additional metrics available in the SMSreg included Multiple Sclerosis Impact Scale–29 (MSIS-29), Expanded Disability Status Scale (EDSS), Symbol Digit Modalities Test (SDMT) scores, and relapses. All tests were assessed along with the EQ-5D on an approximately annual basis, coinciding with an individual's neurology clinic visit. The MSIS-29 is a patient-reported questionnaire that measures the effect of MS based on a set of 29 questions in terms of physical (20 items) and psychological health (9 items), where each question has 5 response levels (not at all, a little, moderately, quite a bit, extremely).^10^ Cumulative scores are generated separately, and transformed on a scale of 0–100, with 0 representing no perceived disability and 100 representing severe disability.^10^ The EDSS is the most commonly used measure of neurologic disability in MS, and is completed by the neurologist as a component of routine clinical care.^11^ We explored the EDSS as a continuous outcome, and categorized as mild (0.0–2.5), moderate (3.0–5.5), and severe (6.0+) neurologic disability. The SDMT is a validated and objective measure of information processing efficiency in MS.^12^ Scores range from 0 to 120, with higher scores indicating greater information-processing efficiency.

Clinical relapses are recorded in the SMSreg, and defined as the acute appearance of neurologic disturbance, lasting ≥24 hours, unrelated to fever or infection.^13^ Persons were considered actively relapsing if they had an EQ-5D assessment within 90 days of a relapse onset. Information on DMT use was collected prospectively and included product name and start and stop dates. DMTs were classified as first-line (interferon-β, glatiramer acetate, teriflunomide, or dimethyl fumarate) and second-line (fingolimod, daclizumab, rituximab, mitoxantrone, and natalizumab) based on prescribing regulations in Sweden. Complete data were available from the SMSreg until February 24, 2019.

### Statistical analyses

Clinical and demographic characteristics and patient-reported outcomes were summarized using frequency (%), mean (SD), or median (interquartile range [IQR]), based on the distribution of the data, and compared between POMS and AOMS using the Pearson χ^2^ or Fisher exact test for categorical variables, and the Student *t* test or Wilcoxon rank-sum test for continuous variables. Cell sizes less than 5 were suppressed to protect confidentiality.

The principal outcomes of interest were the 5 EQ-5D dimensions and the EQ-VAS score. The 5 EQ-5D dimensions were transformed such that “moderate and extreme problems” were combined as “any problems” due to the small cell sizes in the category of “extreme problems.” Reporting “any problems” was compared to “no problems” using logistic mixed-effects models between POMS and AOMS (reference cohort). Mixed-effects allowed for the incorporation of all available scores contributed by an individual, while accounting for the clustering within persons. All analyses were adjusted for sex and time-varying age at assessment, disease course, and DMT exposure. Results were reported as odds ratios (ORs) with 95% confidence intervals (CIs).

The EQ-VAS was selected as an outcome because it is a comprehensive measure of QoL, defined by the individual, as opposed to a summary score based on preselected dimensions of health.^9^ It was modeled as a continuous variable using mixed-effects multivariable linear regression. We compared the EQ-VAS score between patients with POMS and patients with AOMS, and separately assessed the patients with POMS and patients with AOMS to explore potential predictor variables in each cohort. First, we explored the individual effect of each of the 5 health dimensions captured in the EQ-5D on the EQ-VAS at each visit. We then examined sex, age at onset, and region of Sweden (modeled as South, Central, or North Sweden) as fixed variables. Age at assessment, disease duration at assessment, disease course at assessment (modeled as progressive or relapsing), MSIS-29 physical and psychological score, EDSS, SDMT, and DMT exposure were assessed as time-varying covariates. MSIS-29, EDSS, and SDMT scores were included if they were collected on the same day of the EQ-5D assessment or within the previous year. DMT exposure was modeled as first-line therapy or second-line therapy vs no treatment. Persons were considered first- or second-line DMT exposed if they were receiving that drug at the time of the EQ-5D assessment. Covariates were selected based on clinical importance (age and sex) or statistical significance in univariate analyses. The MSIS-29 physical score and EDSS were not included in the same model, given how closely they reflect one another.^14^ The most parsimonious multivariable model was chosen by means of the Akaike Information Criterion.^15^ Results were presented as β-coefficients with 95% CI. Linear mixed model assumptions were verified using QQ plots to test for a normal distribution, and plotting residuals vs fitted values, for linearity and constant variance.

Finally, we modeled the change in EQ-VAS score between visits (including all available visits) using linear mixed effects models to better estimate within-person effects over time.^16^ The dependent variable in these models was the change in EQ-VAS score between visits, and potential predictors included sex, disease course, baseline age, baseline EQ-VAS, and change in EDSS, SDMT, MSIS-29 physical or psychological scores, and time between visits. Statistical analyses were performed using R Version 3.4.3 (Vienna, Austria; R-project.org/).

### Standard protocol approvals, registrations, and patient consents

The study was approved by the Regional Ethical Review Board of Stockholm, and informed consent was provided from patients for the collection of their clinical information.

### Data availability

Data related to the current article are available from Jan Hillert, Karolinska Institutet. To share data from the Swedish MS Registry, a data transfer agreement needs to be completed between Karolinska Institutet and the institution requesting data access. This is in accordance with the data protection legislation in Europe (General Data Protection Regulation). Persons interested in obtaining access to the data should contact Jan Hillert (jan.hillert@ki.se).

## Results

Of 6,722 persons with at least one EQ-5D in the SMSreg, 5,094 met inclusion criteria, of whom 354 (6.9%) had their MS onset in childhood. A total of 21,357 unique EQ-5D assessments were recorded between 2011 and 2019. The median age at onset was 16.3 years (IQR 14.4–17.3) for POMS and 28.9 years (IQR 24.2–34.7) for AOMS. At baseline, patients with POMS were 10 years younger, on average, than the patients with AOMS (27.4 vs 36.9 years). Patients in the POMS group were more likely to have been exposed to a second-line DMT, live in the north of Sweden, and have a higher mean SDMT at baseline than the AOMS group (*p* < 0.05 for all). The sex ratio, disease course, baseline EQ-VAS, EDSS, MSIS-29 physical and psychological scores, and number of relapses during follow-up were all comparable between groups (table 1, unadjusted for age).

**Table 1:**
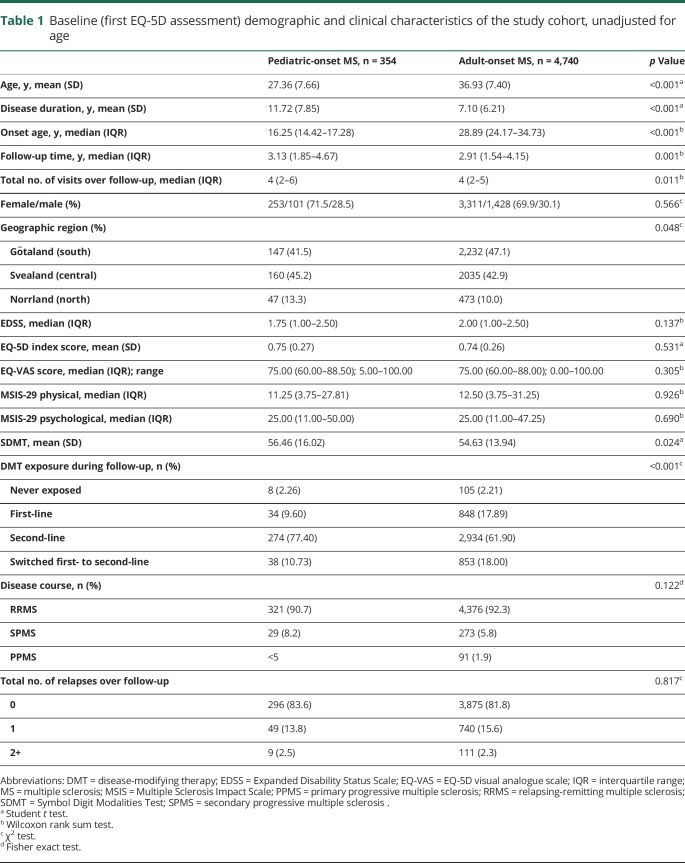
Baseline (first EQ-5D assessment) demographic and clinical characteristics of the study cohort, unadjusted for age

The median EQ-VAS score over the full follow-up was 76 (IQR 60–89), and the mean (SD) was 72.1 (20.3) for the full cohort. A total of 466 (9.14%) persons reported having a “best imaginable health state” on the EQ-VAS (score of 100), and 11 (0.22%) reported a “worst imaginable health state” (score of 0) at least once during follow-up. Less than one-third of the cohort (26.2%) reported problems with mobility at baseline. Few persons reported problems with self-care (5.5%). About one-third (30.7%) reported problems with usual activities, while 55.6% reported pain/discomfort, and 53.7% reported anxiety/depression (table 2).

**Table 2:**
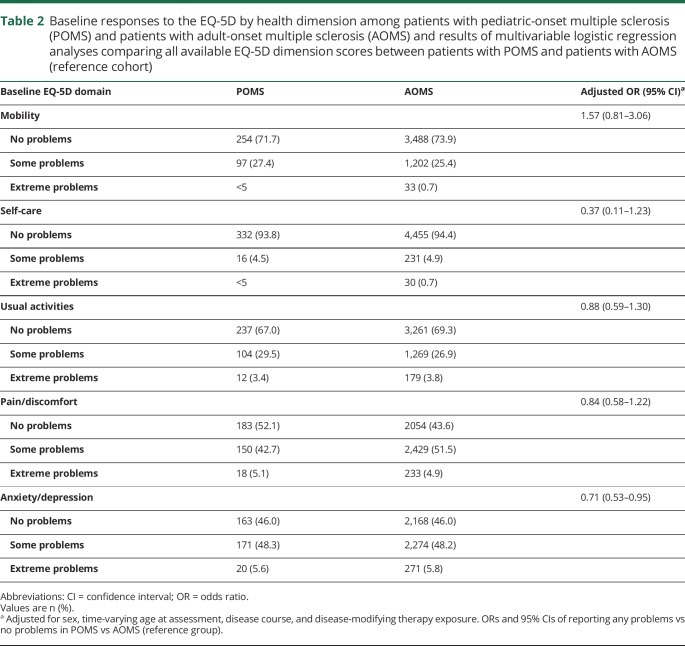
Baseline responses to the EQ-5D by health dimension among patients with pediatric-onset multiple sclerosis (POMS) and patients with adult-onset multiple sclerosis (AOMS) and results of multivariable logistic regression analyses comparing all available EQ-5D dimension scores between patients with POMS and patients with AOMS (reference cohort)

### Differences in EQ-5D dimensions and EQ-VAS between POMS and AOMS

Over the full follow-up, patients with POMS were less likely than patients with AOMS to report anxiety/depression (any problems vs no problems; OR 0.71; 95% CI 0.53–0.95) following adjustment for age, sex, disease course, and DMT exposure. There were no differences between groups in the other 4 EQ-5D dimensions (table 2).

There were no differences in EQ-VAS score between patients with POMS and patients with AOMS in an unadjusted (univariate) mixed effects analysis (β-coefficient [AOMS as reference group]: 1.28; 95% CI −0.57 to 3.13), nor in an analysis adjusted for sex, age, disease course, and DMT exposure (β-coefficient: 0.99; 95% CI −0.89 to 2.87).

### Determinants of QoL in POMS

In univariate linear longitudinal analyses, all 5 dimensions of health were significantly associated with the EQ-VAS in both POMS and AOMS, with effects ranging from −21.2 to −12.0 for “any problems” vs “no problems.” Onset age, age at assessment, and disease duration at assessment were not associated with the EQ-VAS score among patients with POMS (table 3). Women with POMS had significantly lower EQ-VAS scores than men, as did persons living in central and northern Sweden, relative to southern Sweden. Persons with moderate (EDSS ≥3.0 and <6.0) or severe disability on the EDSS (6.0+) reported lower EQ-VAS scores than persons with mild disability (EDSS <3.0). Higher SDMT scores were associated with higher EQ-VAS scores, while increases on either MSIS subscale were associated with lower EQ-VAS scores. Both first- and second-line DMT exposure was associated with higher EQ-VAS score compared to no therapy. Relative to periods of remission, actively relapsing was associated with lower EQ-VAS scores (table 4). The best-fitting adjusted model contained sex, age at assessment, EDSS, SDMT, MSIS-29 psychological score, and DMT exposure. EDSS significantly contributed to EQ-VAS score, as did the SDMT, the psychological effect of MS, and exposure to first-line, but not second-line DMTs (table 5).

**Table 3:**
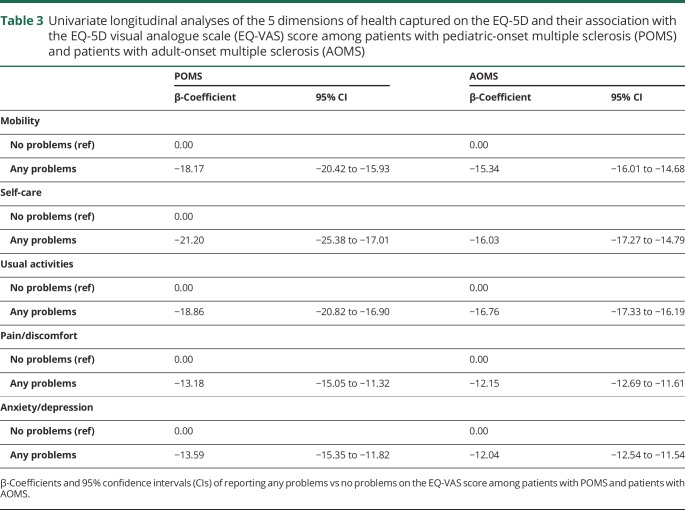
Univariate longitudinal analyses of the 5 dimensions of health captured on the EQ-5D and their association with the EQ-5D visual analogue scale (EQ-VAS) score among patients with pediatric-onset multiple sclerosis (POMS) and patients with adult-onset multiple sclerosis (AOMS)

**Table 4:**
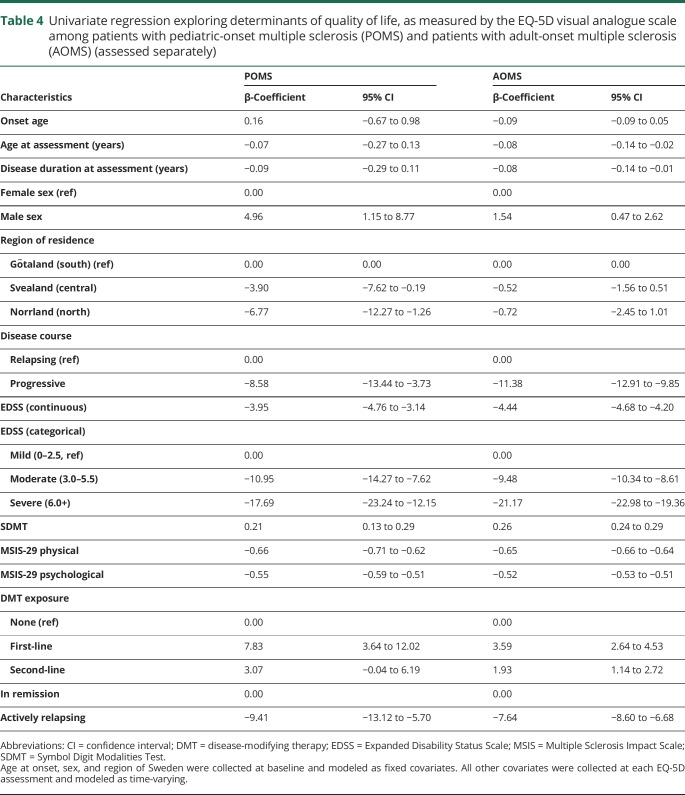
Univariate regression exploring determinants of quality of life, as measured by the EQ-5D visual analogue scale among patients with pediatric-onset multiple sclerosis (POMS) and patients with adult-onset multiple sclerosis (AOMS) (assessed separately)

**Table 5:**
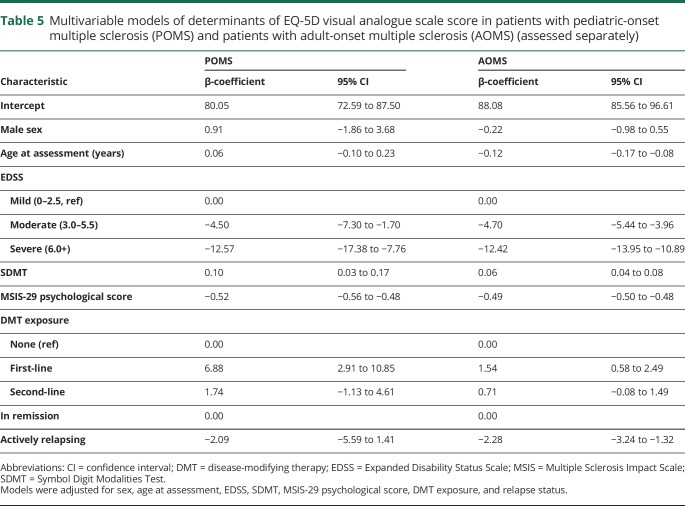
Multivariable models of determinants of EQ-5D visual analogue scale score in patients with pediatric-onset multiple sclerosis (POMS) and patients with adult-onset multiple sclerosis (AOMS) (assessed separately)

### Determinants of QoL in AOMS

Higher onset age, age at assessment, and disease duration at assessment were all associated with lower scores on the EQ-VAS (table 3). Women with AOMS had significantly lower EQ-VAS scores than men. Persons who lived in central Sweden reported lower EQ-VAS scores than persons living in southern Sweden. Moderate or severe disability on the EDSS was associated with lower EQ-VAS scores, while increased information processing efficiency on the SDMT, and lower MSIS-physical and psychological, were each independently associated with a higher EQ-VAS score. First- and second-line DMT exposure were associated with an increase on the EQ-VAS compared to receiving no therapy. Actively relapsing was associated with significantly lower EQ-VAS scores (table 4). In the fully adjusted model, moderate and severe EDSS, increased psychological effect of MS, and experiencing a relapse contributed to a lower EQ-VAS score, while exposure to first-line DMTs and a higher SDMT score contributed to higher EQ-VAS scores (table 5).

### Determinants of change in QoL

There was no difference in change over time between groups (β-coefficient for POMS vs AOMS: −0.40 (95% CI −1.49 to 0.69), following adjustment for sex, age, disease course, DMT exposure, and baseline EQ-VAS score. In the POMS group, significant determinants of decreasing EQ-VAS score were higher baseline EQ-VAS score and increasing MSIS-29 psychological score (table 6), while male sex was associated with increasing EQ-VAS. Among the AOMS cohort, higher baseline EQ-VAS score, increases in EDSS and MSIS-29 psychological score, and transitioning from remission to relapse led to reductions on the EQ-VAS. Exposure to a first-line DMT and improvement on the SDMT contributed to improved EQ-VAS (table 6).

**Table 6:**
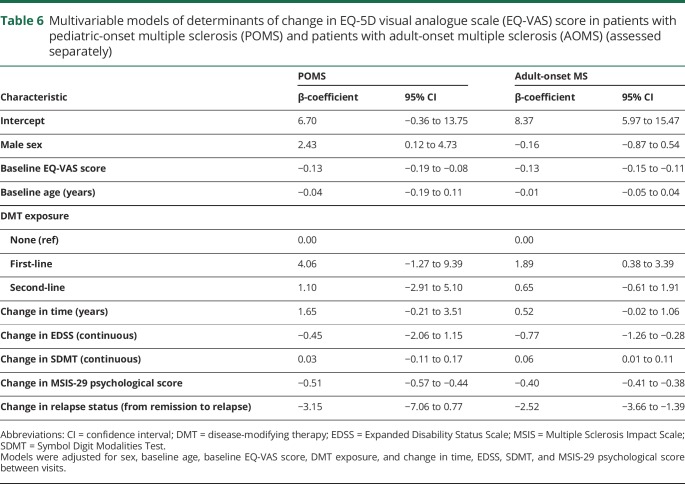
Multivariable models of determinants of change in EQ-5D visual analogue scale (EQ-VAS) score in patients with pediatric-onset multiple sclerosis (POMS) and patients with adult-onset multiple sclerosis (AOMS) (assessed separately)

## Discussion

This was a nationwide, longitudinal cohort study of QoL among persons with POMS and AOMS. At baseline, problems with pain/discomfort or anxiety/depression were reported in nearly half of the cases, while detriments to mobility and usual activities were noted among over one-quarter. Fewer than 10% of the cohort reported any problems with self-care. We found no difference in overall QoL (measured by EQ-VAS) between persons with AOMS and persons with POMS, but those with POMS were less likely to report anxiety/depression over the full follow-up period. In multivariable analyses, similar determinants of QoL were identified in both groups. Experiencing an acute relapse, severe neurologic disability on the EDSS, and high psychological effect of MS (as measured by the MSIS-29 psychological scale) were consistently associated with lower QoL, while higher information processing efficiency (SDMT score) and exposure to first-line DMTs were associated with higher QoL scores. While there was minimal change in QoL over time for the entire cohort, worsening score on the MSIS-29 psychological score was associated with worsening QoL in both groups.

An individual's health-related QoL is largely influenced by their perspective of their position in life in the context of the culture in which they live.^17^ It can also be thought of as “the extent to which an individual's hopes and ambitions are matched and fulfilled by experience.”^18^ In the context of chronic illness, this can mean that a person's perceived QoL is in a dynamic state, constantly adapting to the current stage of disability. Given the known consequences of POMS on physical and cognitive abilities relative to AOMS,^4,19^ it is notable that, overall, patients with POMS did not perceive their health status as significantly worse than did patients with AOMS.

Reporting problems in any of the 5 domains of health collected on the EQ-5D was associated with a lower mean EQ-VAS score. This raises the question of whether specifically targeting these areas could improve QoL. Anxiety or depression were endorsed by over half of the cohort, suggesting that active management of both (pharmacologic or nonpharmacologic) should be a clinical priority, given the high prevalence and potential to improve QoL.

Experiencing an acute relapse and neurologic disability, measured by the EDSS, were both significant contributors to impaired QoL, in line with previous findings.^1,20^ Slowing disability progression and reducing relapse rates have long been goals of MS treatment, and these results suggest that these are meaningful targets. First-line DMT exposure was associated with a higher QoL and improved QoL among patients with AOMS, suggesting that drug treatment may contribute to improvements in QoL. These results should be interpreted cautiously, however. Second-line DMTs were not consistently associated with improved QoL, which suggests that findings may be due to indication bias.^21^ Persons with more severe disease are more likely to be treated with second-line therapies (or switched from first- to second-line therapies), and this disease worsening likely also contributes to worse health-related QoL.

While all of these factors were statistically significant, their clinical significance is less clear. There are, to our knowledge, no previous studies on minimal clinically important differences on the EQ-VAS for this particular patient group. As EQ-VAS represents the respondent's assessment of his or her own health (as opposed to the EQ-5D index value, which is calculated through value sets based on preferences from the general population), we consider EQ-5D VAS scores to be more closely linked to the patient's own perspective of a meaningful change, compared to the index values.

Both psychological effect of MS and detriments to information processing efficiency were consistently associated with lower QoL scores, albeit with small effect sizes. Nonetheless, addressing the psychological effects and cognitive detriments of MS may also be a means of improving QoL, through improved support for these symptoms.

Six identified studies explored QoL among persons with POMS and all employed the Pediatric Quality of Life Inventory.^22–27^ Sample sizes ranged from 41^23^ to 64 MS cases,^24^ and all were completed in the United States^22–24^ or Italy.^25–27^ Similar to our findings, the factor most consistently associated with a worse QoL among patients with POMS during childhood was higher EDSS score.^22–25^ Fatigue^22,24^ was also consistently associated with reduced QoL scores, and one study found that affective disorders were associated with lower QoL, while resilience competence was associated with higher QoL.^26^ We found no effect of DMT exposure on within-person QoL change in POMS, but a single-arm observational study of a self-injecting device for β-interferon reported improvements in QoL from baseline to end of treatment at 52 weeks.^27^

The literature regarding determinants of QoL in patients with AOMS is much larger and has been summarized in a review^1^; consistent predictors of reduced health-related QoL include disability, depression, cognitive impairment, pain, hopelessness, and lack of autonomy and support.^1^ While we did not have information on each of these determinants, our results were consistent with those captured, including impairments to cognition, psychological effect, and physical disability.

Strengths of this study include the use of the large, population-based Swedish MS Registry. The EQ-5D has been described as satisfactory for use within the MS population^28^; it is the most widely used metric of health-related QoL in the QoL and health economics literature,^29^ and it has been used extensively within the field of MS research.^1,30^ Nonetheless, employing the EQ-VAS as the primary outcome may have limited interpretation of findings as a minimal clinically important difference has not been established in the context of MS, which precluded us from commenting on the clinical significance of our results.

The use of objective information on neurologic disability and information processing efficiency, and the availability of serially collected measures of QoL enabling us to evaluate the individual-level effects on changing QoL over time, were further strengths of the study. Limitations include a lack of information on other factors that likely contribute to QoL, such as fatigue, body mass index, and work and family situation. Though this represents a large cohort of persons with POMS given the rarity of the condition, we may still have been underpowered to detect differences within this group. This may have contributed to some of the differences observed between POMS and AOMS following stratification by group. For instance, effect sizes were often similar between groups, but the wide CIs among POMS meant that statistical significance was not achieved. It is possible that a larger sample size, followed over a longer period of time, may have elicited different findings. Few persons with POMS reported having “extreme” problems on the EQ-5D domains, which precluded us from exploring this category as an outcome. All persons in this study were followed in an outpatient neurology clinic, and most were receiving a DMT. It is possible that these results are not generalizable to the wider MS population of untreated persons, or persons who do not attend clinic.^31^ Finally, Sweden is a country that consistently scores high on QoL metrics at the population level^32^; these results may not be generalizable to persons living in other nations.

Overall QoL among persons with MS did not appear to be influenced by having a pediatric onset of disease. Severe neurologic disability, experiencing a relapse, increased psychological effect of MS, and reduced information processing efficiency were consistent and significant determinants of lower QoL in both POMS and AOMS. The ultimate aim of chronic disease treatment is to improve longevity and QoL. Regardless of onset age, this study highlights that impairments to QoL are common in MS, and that management of disease should incorporate efforts to improve well-being and QoL. These findings should be utilized to assist health care providers in identifying persons who may be at risk of declines in QoL.

## Glossary

AOMS: adult-onset multiple sclerosis
CI: confidence interval
DMT: disease-modifying therapy
EDSS: Expanded Disability Status Scale
EQ-VAS: EQ-5D visual analogue scale
IQR: interquartile range
MS: multiple sclerosis
MSIS-29: Multiple Sclerosis Impact Scale–29
OR: odds ratio
POMS: pediatric-onset multiple sclerosis
QoL: quality of life
SDMT: Symbol Digit Modalities Test
SMSreg: Swedish MS Registry
